# Characteristic Analysis of Homo- and Heterodimeric Complexes of Human Mitochondrial Pyruvate Carrier Related to Metabolic Diseases

**DOI:** 10.3390/ijms21093403

**Published:** 2020-05-11

**Authors:** Jinyi Lee, Zeyu Jin, Donghan Lee, Ji-Hye Yun, Weontae Lee

**Affiliations:** 1 Department of Biochemistry, College of Life Science & Biotechnology, Yonsei University, Seoul 03722, Korea; jylee@spin.yonsei.ac.kr (J.L.); zyjin@spin.yonsei.ac.kr (Z.J.); 2 Department of Medicine, James Graham Brown Cancer Center, University of Louisville, Louisville, KY 40292, USA; donghan.lee@louisville.edu

**Keywords:** human mitochondrial pyruvate carrier, hetero-complex, cellular homeostasis, oligomerization

## Abstract

Human mitochondrial pyruvate carriers (hMPCs), which are required for the uptake of pyruvate into mitochondria, are associated with several metabolic diseases, including type 2 diabetes and various cancers. Yeast MPC was recently demonstrated to form a functional unit of heterodimers. However, human MPC-1 (hMPC-1) and MPC-2 (hMPC-2) have not yet been individually isolated for their detailed characterization, in particular in terms of their structural and functional properties, namely, whether they exist as homo- or heterodimers. In this study, hMPC-1 and hMPC-2 were successfully isolated in micelles and they formed stable homodimers. However, the heterodimer state was found to be dominant when both hMPC-1 and hMPC-2 were present. In addition, as heterodimers, the molecules exhibited a higher binding capacity to both substrates and inhibitors, together with a larger structural stability than when they existed as homodimers. Taken together, our results demonstrated that the hetero-dimerization of hMPCs is the main functional unit of the pyruvate metabolism, providing a structural insight into the transport mechanisms of hMPCs.

## 1. Introduction

As the final product of glycolysis, pyruvate is essential for numerous aspects of the human metabolism [1]. It is necessary for the generation of ATP and drives several major biosynthetic pathways in mitochondria [1]. Many studies of the pyruvate metabolism have evaluated the correlations between diseases and pyruvate flow [1,2,3,4]. The pyruvate branch point, which controls the flow of pyruvate between oxidative and fermentative routes, is central to cellular homeostasis. Although the transport of pyruvate into mitochondria has been studied for decades [5,6], mitochondrial pyruvate carriers (MPCs), which play key roles in pyruvate uptake, have been discovered only recently [7,8]. MPCs regulate the uptake of pyruvate from the mitochondrial intermembrane space into the mitochondrial matrix, the dysfunction of which can lead to the development of metabolic diseases [9]. The discovery of MPCs has led to a number of molecular genetic studies, including those on numerous cellular processes controlled by pyruvate uptake.

Human MPCs (hMPCs) include two protomers, hMPC-1 and hMPC-2, which are essential for pyruvate transport [2,7]. However, the homo- and heterotypic assembly and stoichiometry of the two protomers is not yet fully understood [1,10]. Very recently, structural analyses demonstrated that the functional units of yeast MPC are heterodimers [11]. In a previous study, hMPCs were co-expressed and purified using a yeast expression system, and although no substantial complex formation was observed, the individual function of hMPC-2 as an autonomous pyruvate transporter was demonstrated [12]. Subsequently, the molecular characterization of the homo- and heterotypic assemblies of hMPCs in vitro is necessary to improve our understanding of these molecules.

Recently, several studies on MPCs have focused on the individual activities of homotypic as well as heterotypic MPCs. As a result, the downregulation or deletion of MPC-1 was found to be associated with various types of cancer, while MPC-2 was found to play an important role in the control of hepatic gluconeogenesis [13,14,15]. Thus, the different expression levels and activities of individual MPCs and the ratio of heterotypic MPC formation are associated with differences in cellular metabolism. Moreover, the dysfunction of MPCs has been associated with various diseases, including cancer, Alzheimer’s disease, and type 2 diabetes, caused by abnormal metabolic activities. As a result, MPCs represent a potential target molecule for the development of therapeutic drugs [16,17,18,19,20].

Several compounds have been shown to inhibit MPC activity [21,22,23,24]. For example, thiazolidinediones (TZD), a group of oral anti-diabetic drugs, act as ligands for PPARγ (peroxisome proliferator-activated receptor-gamma) [3,25,26] and inhibit MPC activity at clinically relevant concentrations [27]. α-cyano-β-(1-phenylindol-3-yl)-acrylate (UK5099), an efficient blocker of MPC, inhibits pyruvate-dependent oxygen consumption and pyruvate mitochondrial transportation in vitro [2,22,28].

This study reported the successful expression and purification of individual hMPC-1 and hMPC-2 using the baculovirus expression system in *Sf9*. Moreover, the homotypic or heterotypic assembly of recombinant hMPC-1 and hMPC-2 and their biochemical properties, including thermal stability, folding states, and binding affinity, of the homo- and heterotypic conformations of MPCs in vitro were demonstrated. Our findings suggested that the heterodimer of hMPCs is structurally more stable and efficient conformation, consistent with recent research [11,12]. These results provide an insight into the conformations and functional correlations of hMPCs and provide a basis for the characterization of the three-dimensional structure of hMPCs for drug design.

## 2. Results

### 2.1. Expression and Purification of hMPCs

**Figure 1:**
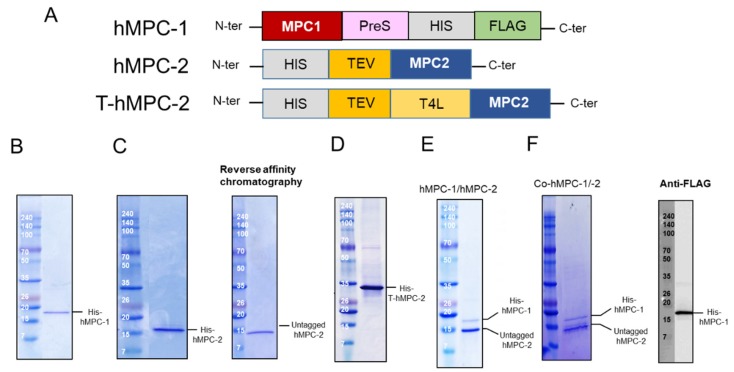
Construct design and purification of human mitochondrial pyruvate carriers (hMPCs). (**A**) Schematic diagram of designed hMPC constructs. (**B**–**D**) Purification profiles of (**B**) His-hMPC-1, (**C**) His-hMPC-2 (left) and untagged hMPC-2 (right), and (**D**) His-T-hMPC-2. The N-terminal tags were removed from hMPC-2 by Tobacco Etch Virus (TEV). Un-tagged hMPC-2 (14.3 kDa) was isolated after reversed affinity column. The recombinant proteins were visualized by Coomassie blue staining in SDS-PAGE after metal affinity purification. (**E**) His-hMPC-1 (15.9 kDa) and untagged hMPC-2 (14.3 kDa) after the FLAG-resin column was co-eluted and confirmed by SDS-PAGE. (**F**) The co-expressed proteins, co-hMPC-1/-2, were confirmed by Coomassie blue staining in SDS-PAGE after purification. After TEV cleavage reaction, His-hMPC-1 and untagged hMPC-2 was co-eluted in the FLAG-resin column. hMPC-1 was confirmed by anti-FLAG-tag immunoblot analysis.

Although MPC-1 and MPC-2 are generally considered to form heterotypic complexes, there is little structural evidence to support this in vitro [4,10]. hMPCs were recently characterized in vitro, however, more information about their formation of heterotypic complexes is needed, since previous studies have mainly focused on the homotypic complex of hMPC-2, rather than its formation of a heterotypic complex with hMPC-1 [12]. In the case of hMPC-1, the N-terminal tags, including the 10× His-tag, FLAG tag, and PreScission enzyme recognition sequence, were moved to the C-terminal region for protein expression (Figure 1A). None of the hMPC-1 constructs containing N-terminal tags expressed well. To determine the structural and biochemical properties of the heterotypic complex of hMPCs in vitro, two constructs of hMPC-2 were designed and evaluated for their size distribution using size-exclusion gel chromatography (SEC). The first, labeled hMPC-2, contained an influenza hemagglutinin (HA) tag, 10× His-tag, and Tobacco Etch Virus (TEV) cleavage sequence at the N-terminus. The other, labeled T-hMPC-2, contained an additional T4 Lysozyme (T4L) sequence after the 10× His-tag (Figure 1A). A modified pFastBac vector comprised of a polyhedrin (PH) promoter and a HA tag was used for all of the constructs to increase expression on the membrane [29,30].

Three constructs, hMPC-1, hMPC-2, and T-hMPC-2, were successfully purified using a TALON affinity column in buffer containing 20 mM HEPES (pH 8), 150 mM NaCl, and 0.05% (*w*/*v*) DDM with 500 mM imidazole (Figure 1B–D). The purified hMPC-1, hMPC-2, and T-hMPC-2 showed a single protein band at the expected molecular weight in SDS-PAGE analysis. For hMPC-2, the sample was treated with TEV enzyme for tag cleavage, after which the untagged protein was isolated using reversed affinity column (Figure 1C). For the hMPC-1/hMPC-2 complex sample, C-terminal tagged hMPC-1 and untagged hMPC-2 were used. After mixing hMPC-1 and hMPC-2 at a 1:1 molar ratio, FLAG-resin column work were performed. hMPC-2 was found to co-elute with hMPC-1, indicating that they interacted to form a stable complex (Figure 1E).

hMPC-1 and hMPC-2 (co-hMPC-1/-2) were co-expressed using a co-infection method in Sf9 cells, the co-eluted MPCs was confirmed after purification using both Ni-NTA and FLAG affinity column works (Figure 1F). In our result, since the intensities of the purified hMPC-1 was relatively weaker than those of the purified hMPC-2, we verified the hMPC-1 band using a FLAG-antibody, which does not exist in hMPC-2 (Figure 1F).

**Figure 2:**
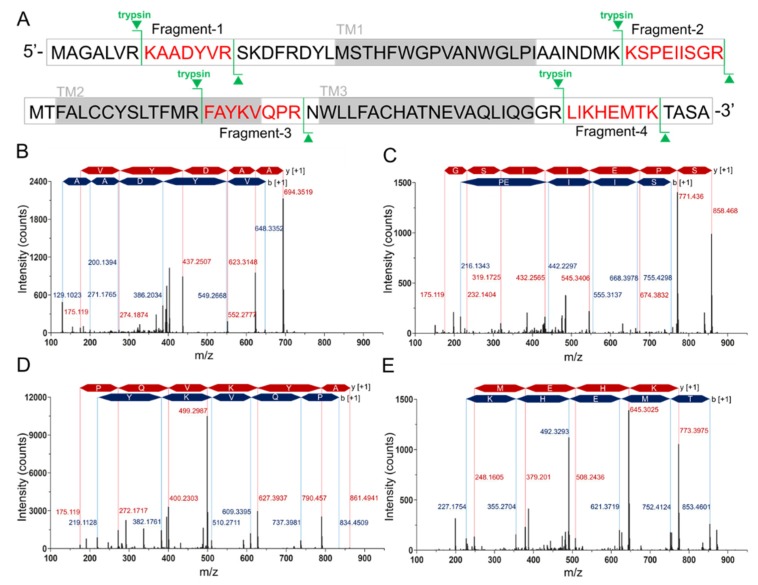
Tryptic peptide mapping of purified hMPC-1 by nano-LC-LTQ-Orbitrap-MS spectrum. (**A**) Sequence coverage of identified tryptic peptide fragments of hMPC-1. The peptide fragments identified in the LC-LTQ mass spectrum are highlighted in red. The trypsin digestion sites are marked in green lines. Gray boxes denote the transmembrane region. (**B–E**) The nano-LC-LTQ-Orbitrap-MS spectrum of tryptic peptide hMPC-1 fragments. The identified tryptic fragments sequence was as follows: (**B**) KAADYVR, (**C**) KSPEIISGR, (**D**) FAYKVQPR, and (**E**) LIKHEMTK. The peaks of the b-ion and y-ion are marked in blue and red together with identified amino acid sequences. The m/z of the identified peaks is labeled with blue and red characters.

Since the difficulty of the protein expression and the similar molecular weight of hMPC-1 than hMPC-2, we further analyzed hMPC-1 through the liquid chromatography-mass spectrometry (LC-MS) (Figure 2). In the tryptic peptide mapping, four trypsin digested peptide fragments such as KAADYVR (Figure 2B), KSPEIISGR (Figure 2C), FAYKVQPR (Figure 2D), and LIKHEMTK (Figure 2E) were seen. Sequence coverage is around 25% due to the limitation of the cleavage site of the trypsin. The purity of the protein was over 95% and no further peptide derived from other impurities identified. Non-specific glycosylation modification often occurs during protein expression in sf9 cells, which may affect the function of the target protein. In our experiments, there was no evidence of the glycosylation modification in the purified hMPCs from sf9 cell.

The final yields of hMPC-1, hMPC-2, and T-hMPC-2 were approximately 0.9 mg, 0.8 mg, and 1.25 mg per liter, respectively. These results demonstrate the successful expression and purification of hMPCs on a large scale. These samples were then used for further characterization in vitro.

### 2.2. Homo- and Hetero-Complex Formation of hMPC-1 and hMPC-2

**Figure 3:**
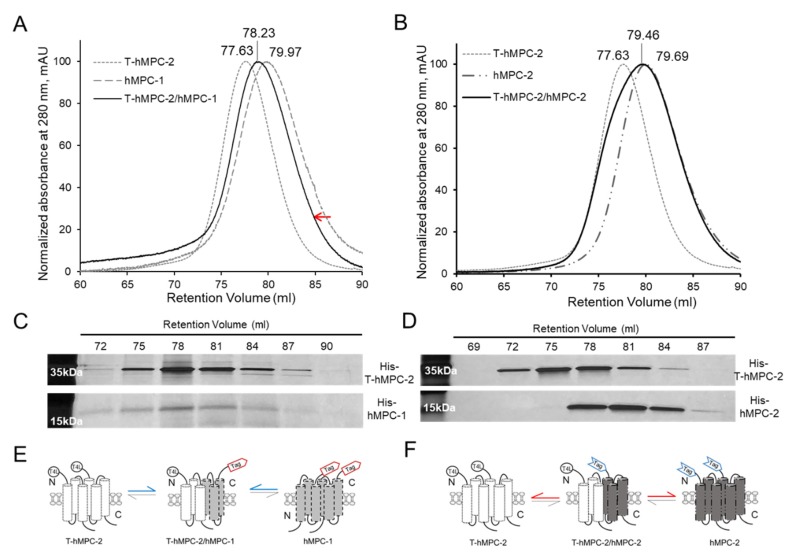
Hetero-complex formation between hMPC-1 and hMPC-2. (**A**,**B**) Analytical size-exclusion gel chromatography (aSEC) assay for identifying homo- and hetero-complexes of hMPCs. The monodisperse peaks of hMPC-1, hMPC-2, and T-hMPC-2 are represented by a dark gray dashed line, dash-double dotted line, and light gray dotted line, respectively. Both peaks of T-hMPC-2/hMPC-1 and T-hMPC-2/hMPC-2 are shown as a black line. The retention volumes of hMPC-1, hMPC-2, T-hMPC-2, T-hMPC-2/hMPC-1, and T-hMPC-2/hMPC-2 were 79.97 mL, 79.69 mL, 77.63 mL, 78.23 mL, and 79.46 mL, respectively. All aSEC analyses were performed using HiLoad Superdex 200 PG equilibrated with buffer containing 50 mM Tris-HCl (pH 7), 300 mM NaCl, and 0.05% (*w*/*v*) DDM. Absorbance was monitored at 280 nm. The peak shifting indicates by a red arrow. (**C**,**D**) SDS-PAGE analysis of eluted proteins across the retention volume (mL). The peaks containing T-hMPC-2/hMPC-1 (**C**) and T-hMPC-2/hMPC-2 (**D**) were isolated by each fraction separately, and protein contents were analyzed by SDS-PAGE. (**E**,**F**) Schematic diagram of the proposed process for the homo- and hetero-complexes of hMPC-1 and hMPC-2 based on the experimental setup. The higher strength of binding and dissociation constants about MPCs heterodimerization was highlighted in blue and red arrows, respectively.

Since hMPCs have not yet been individually purified in previous studies, little is known about the interaction between hMPC-1 and hMPC-2 during hetero-complex formation in vitro. Here, analytical size-exclusion gel chromatography (aSEC) was used to characterize the homo- and hetero-complex formation of hMPC-1 or hMPC-2 mixed with T-hMPC-2 in vitro (Figure 3A,B). T-hMPC-2 was used to differentiate between the molecular weight of the hMPCs during heterodimer complex formation since the molecular weights of hMPC-1 and hMPC-2 are very similar. The N-terminal fusion T4L is a well-validated and widely used fusion protein, not affecting the functional and structural modification of membrane protein. The retention volume of the T-hMPC-2 homo-dimer was 77.63 mL, while those of the hMPC-1 and hMPC-2 homodimers were 79.97 and 79.69 mL, respectively. The T-hMPC-2 and hMPC-1 mixture was eluted in 78.23 mL with a monodisperse peak by aSEC (Figure 3A), whereas the T-hMPC-2 and hMPC-2 mixture was not detected in the monodisperse form (Figure 3B). Based on the peak fraction obtained by SDS-PAGE, T-hMPC-2 and hMPC-1 were found to form a stable heterodimer eluted at the same retention volume (Figure 3C), whereas T-hMPC-2 and hMPC-2 eluted separately at different retention volumes (Figure 3D). Even though some fraction volumes between 78 mL and 81 mL overlapped, the data were interpreted based on the maximum intensity of each fraction. The protein signal of hMPC-1 tends to be weaker than T-hMPC-2 and a similar observation was made in co-eluted hMPC-1/hMPC-2 and co-hMPC-1/-2 (Figure 1E,F). This observation may be attributed to the low aromatic residue content in hMPC-1 (Figure S1). Although the weak point of our SEC profile was the fact that the calculation of the heterodimeric proportions was not highly accurate, the SEC profile of T4L-hMPC- 2 mixed with hMPC-1 and hMPC-2 in the same environment was clearly different, which may be related to the equilibrium dissociation rate constant of the hMPCs. When hMPC-1 and hMPC-2 co-existed, the equilibrium dissociation rate constant of heterodimer seems to be lower than that of homodimer formation of hMPC-1 and hMPC-2. Thus, heterodimer formation was dominant than that of each homodimer whereas hMPC-2 and T4L-hMPC-2 were stable, not affecting each other, due to their transmembrane regions having a dimerization region that was virtually identical (Figure 3E,F).

### 2.3. The hMPC-1/hMPC-2 Heterodimer Has a Greater Binding Efficiency for Substrate and Inhibitors Than Homodimers

**Figure 4:**
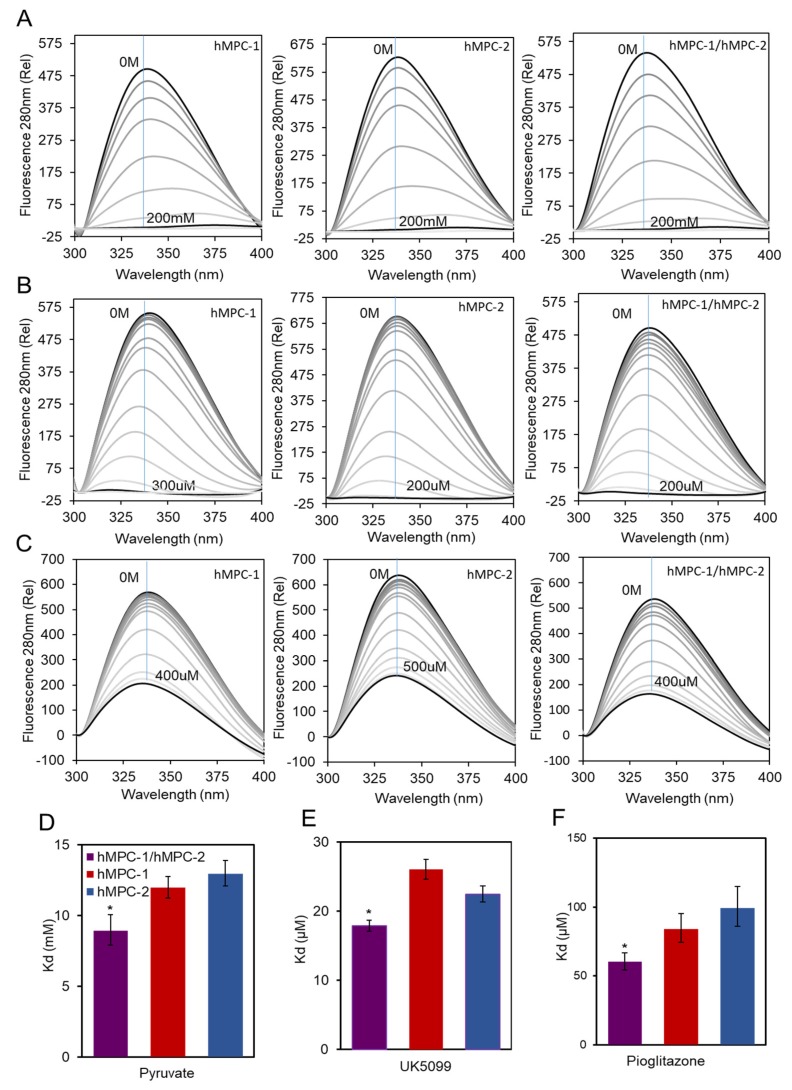
Molecular interaction of hMPCs with substrates and inhibitors. Fluorescence spectrum of the hMPC-1, hMPC-2, and hMPC-1/hMPC-2 on the titrating of (**A**) pyruvate up to 200 mM, (**B**) UK6099 up to 200 or 300 µM, and (**C**) pioglitazone up to 400 or 500 µM. The blue line indicates the intensity values in the 338nm wavelength of each spectrum used for calculating the dissociation constants. The equilibrium dissociation constants (K_d_) of hMPCs were calculated using a fluorescence quenching assay with (**D**) pyruvate, (**E**) UK6099, and (**F**) pioglitazone. The K_d_ values for hMPC-1 (red), hMPC-2 (blue), and hMPC-1/hMPC-2 (purple) for each ligand are summarized in Table S1. Errors were determined based on three replicated experiments. The error bars represent the standard deviation (SD), *n* = 3. * *p* = 0.05.

In vitro fluorescence quenching was performed to measure the binding affinity of the purified hMPCs to their ligands, such as the pyruvate substrate and inhibitors, to validate their function (Figure 4). First, the equilibrium dissociation constants (K_d_) of the hMPCs to pyruvate substrates were measured (Figure 4A,D). The binding affinity between the hMPC-1/hMPC-2 hetero-complex and pyruvate substrate was 1.5-fold higher than that of the homo-complex, suggesting possibilities of the functional modulation of pyruvate transport efficiencies in vivo (Table S1).

Although the homo-complex of hMPCs exhibited a lower binding affinity than that of the hetero-complex, it seems to have its own function since the differences in terms of the binding affinity of the homo- and hetero-complexes were not extreme. Our results correlate with those of previous studies, indicating that the homo-complex of MPCs still reacts with pyruvate [12]. The same experiments were performed using MPC inhibitors to evaluate the binding affinity of the homo- and hetero-complexes of hMPCs. The most notable inhibitors, UK5099 and pioglitazone, were selected. The efficacy of UK5099, an MPC-specific inhibitor, has been demonstrated by several in vivo studies; as such, it is a drug candidate for the treatment of numerous diseases [20]. Pioglitazone, a TZD, is a widely used insulin-sensitizer [26]. The binding affinities of the hMPC-1 and hMPC-2 homo-complexes for both ligands were increased by hetero-complex formation (Figure 4B,C,E,F). This was consistent with the results for pyruvate binding in terms of tendency (Figure 4A,D). The binding affinities with inhibitors were stronger (K_d_ values of μM) than that with pyruvate (K_d_ values of mM) and the homo-complexes of hMPCs still showed strong binding to inhibitors (Table S1). These results indicate that inhibitor binding is not specific to specific protomers of hMPCs. Overall, the affinity of the binding to each substrate was lowest for the homo-complex of hMPC-2, except for binding to UK5099, with highest binding for the hMPC-1/hMPC-2 hetero-complex. These results indicate that the hetero-complex of hMPCs is a more efficient unit compared with the homo-complex during the binding process with ligands (Table S1).

This is the first analysis of binding affinities in vitro using purified hMPCs and their substrates. Recent reports have suggested insights into the functional units of MPCs. In yeast, heterodimers cannot occur in the absence of MPC-1, and the homodimers of MPC-2 and MPC-3 are functionally inactive [11]. In addition, another study reported that human MPCs exist as heterodimers dominantly [7,12].

Our results highlight the differences in biochemical properties by comparing the status of the hMPC-1/hMPC-2 heterodimer complex in vitro, which is considered to be such a functional unit, with that of each monomer. Although tighter binding does not directly explain the functional improvement, our results suggest differences in the binding capacity between hMPC-1, hMPC-2, and hMPC-1/hMPC-2. Our findings suggest that their function may be modulated at the cell level by the formation of a heterodimer between hMPC-1 and hMPC-2.

### 2.4. The hMPC-1/hMPC-2 Heterodimer Has a Greater Stability Than Homodimers

**Figure 5:**
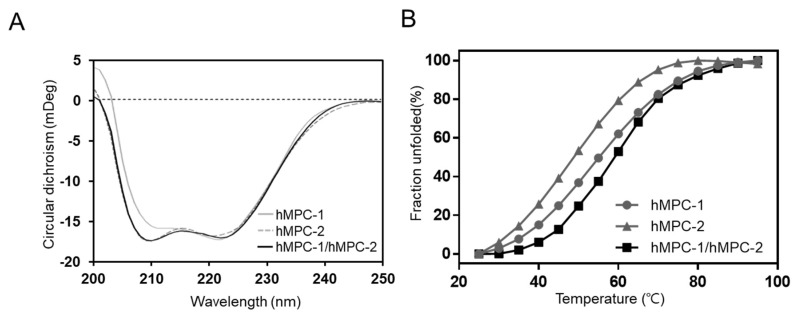
Secondary structure and thermal stability of hMPC-1, hMPC-2, and hMPC-1/hMPC-2. (**A**) Far-UV circular dichroism spectra (200–250 nm) of hMPC-1 (light gray line), hMPC-2 (dark gray dashed line), and hMPC-1/hMPC-2 (black line). (**B**) The melting temperature (*T_m_*) was calculated by the decrease in intensity values at 217 nm in the CD spectrum. *T_m_* values for hMPC-1 (light gray line with circle), hMPC-2 (dark gray line with triangle), and hMPC-1/hMPC-2 (black line with square) were 54.4 ± 0.86 °C, 47.6 ± 1.23 °C, and 58.5 ± 1.33 °C, respectively. All experiments were repeated three times to calculate the standard deviation.

Previous studies have shown that MPCs can exist as functional units not only in heterozygous complexes but also in homozygous complexes [11,12]; accordingly, it is important to analyze their structural properties. Circular dichroism (CD) spectra provided information related to the secondary structure and thermal stability of homo- and heterotypic hMPCs (Figure 5). Purified hMPCs, such as hMPC-1, hMPC-2, and hMPC-1/hMPC-2, clearly showed secondary structural properties of the α-helix in CD spectra. After calculating the contents of the secondary structure, hMPC-1 and hMPC-2 were found to be comprised of 79.8% and 91.5% α-helices, respectively. Although hMPC-1 showed a lower α-helix content than hMPC-2 due to the C-terminal-tags, all hMPCs were stable enough by their native fold in the micelle environment. Moreover, the overall shape of the CD spectra for hMPCs were not markedly different (Figure 5A). Of note, the overall shape of the hMPC-1/hMPC-2 CD spectra were almost identical to that of hMPC-2. However, the thermal stability differed substantially between hMPC-1 and hMPC-2 (Figure 5B). The melting temperature (*T_m_*) of hMPC-1 (54.4 ± 0.86°C) was much higher than that of hMPC-2 (47.6 ± 1.23°C). Moreover, hMPC-1/hMPC-2 showed higher *T_m_* values (58.5 ± 1.33 °C) than hMPC-1.

Our results showed that hMPC-1 and hMPC-2 were individually present as homo-complexes, but preferentially existed in the more stable form of hetero-complexes with another protomer. This is a reasonable explanation for the tendency of MPCs to form hetero-complexes dominantly rather than homo-complexes.

### 2.5. Homology Model for the Homotypic and Heterotypic Conformations of hMPCs

**Figure 6:**
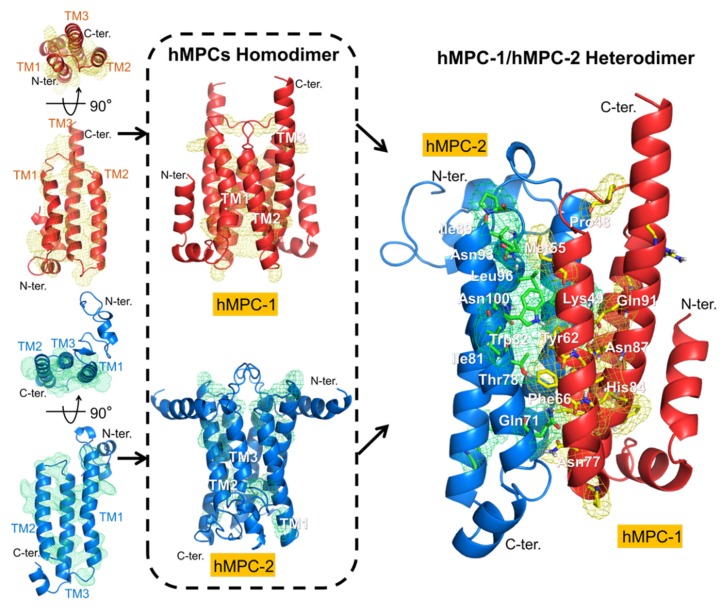
Model structures of homo- and hetero-complexes of hMPCs. Comparison of the structures of the monomeric units of hMPC-1 (red) and hMPC-2 (blue) based on the semisweet transporter are represented by a ribbon model (**left**). Highly conserved residues in hMPC-1 and hMPC-2 are shown by yellow and green mesh. The homodimer (middle) and heterodimer (**right**) structures of hMPC-1 and hMPC-2 generated from their monomeric structures indicate with the black arrows. The highly conserved residues in the transmembrane domain were predicted in a dimeric interface important for the functional pathway. The conserved residues in hMPC-1 and hMPC-2 are highlighted as stick models in yellow and green, respectively.

By aligning the primary sequences of hMPC-1 and hMPC-2 with those of other species (Figure S1), the region of the transmembrane helices related to pyruvate transport activity was found to be highly conserved. The sequence identity in this region between humans and several mammals, including bovines, rats, and mice, was over 90%, while the identity with sequences of eukaryotic species, such as zebrafish, thale cress, *C. elegans*, and yeast, was approximately 30%. Accordingly, the detailed molecular properties and major molecular mechanisms of human-derived MPCs are not limited to humans but may also apply to other species. In particular, the locations of several highly conserved residues in hMPC-1 and hMPC-2 in all species were analyzed using homology modeling (Figure 6).

The homotypic and heterotypic model structures of hMPCs were built using several modeling and refining programs on the GalaxyWEB server with SemiSWEET transporter (PDB code: 4QND) as a template. The homotypic models of hMPC-1 and hMPC-2 were analyzed with respect to the overall topology. The membrane anchoring in the transmembrane regions occurred in opposite directions. The N-terminal region of hMPC-1 was located in the cytosolic region, whereas that of hMPC-2 was located in the extracellular region. When analyzing the internal packing of the hMPC-1 and hMPC-2 model structures, hMPC-1 was found to have a larger protein packing value than hMPC-2 (Figure S2). In addition, by measuring the value of the internal pocket generated by their homodimeric interface, the pocket volume inside the interface of hMPC-1 was found to be much smaller than that of hMPC-2 (Figure S3). These results suggested that hMPC-1 and hMPC-2 may have differences in the structural stability of their three-dimensional packing. In summary, the overall folding of the monomeric and dimeric models of hMPCs suggest that hMPC-1 has a more compact and stable form than hMPC-2 (Figure 6), which is highly consistent with the thermal stability results.

Interestingly, the highly conserved residues, including pro48, Met55, Tyr62, Phe66, Asn77, His84, Asn87, and Gln91 in hMPC1 and Gln71, Thr78, Ile81, Trp82, Ile89, Asn93, Leu96, and Asn100 in hMPC-2, were located on the same surface as their dimeric interface in the homo- and hetero-complexes.

## 3. Discussion

Recently, evidence has suggested that MPCs are good therapeutic targets for neurodegenerative and metabolic diseases, including Parkinson’s disease, cancer, and type 2 diabetes [18,19,31,32]. Moreover, the expression levels of MPC1 and MPC2 differ in various diseases, and the reduced expression of the MPC hetero-complex is associated with unfavorable clinicopathological characteristics [33]. The elucidation of the formation and properties of hetero- or homo-complex structures are very important owing to their direct links to functional and pathological features [33,34]. However, the composition of MPC complexes, oligomer states, and stoichiometry remain a topic of controversy. Despite the whole-body metabolic homeostasis of MPC and its important role in disease, little is known about the relationship between its function and structural conformation.

In this study, the large-scale purification of both hMPC-1 and hMPC-2 was performed for the first time using insect cells. Interestingly, although the same strategy was applied to hMPC-1 and hMPC-2 for high expression, their behaviors were markedly different. hMPC-1 with N-terminal tags was not well expressed, whereas hMPC-2 with N-terminal tags was expressed well in *Sf9*. This suggests that the orientation during membrane anchoring differs [4]. Our model structures of hMPC-1 and hMPC-2 also suggest that they preferentially form a hetero-complex with reverse topology [4,35]. Very recently, hMPC-1 and hMPC-2 were co-expressed and purified in yeast; however, reliable homo- and hetero-complexes were not comprehensively characterized owing to the fact that a co-expression system was studied, rather than individual expression systems [12]. Thus, an advantage of our approach is that hMPC-1 and hMPC-2 were expressed and purified using both single and co-expression systems, with the flexibility to perform accurate comparisons in each experiment. Our results indicated that hMPC-1 is thermodynamically more stable than hMPC-2 and is the main determinant of the formation of hetero-complexes. These results support those reported in previous studies wherein hMPC-1 was found to be a key molecule in mitochondrial respiration, energy generation, various cancers, and type 2 diabetic kidney disease; despite this, hMPC-2 is also important [12,13,36,37,38]. In several studies, both the homo- and heterodimers of hMPCs have been proposed as functional units [11,12]. Our results suggest that the heterodimeric state is the more stable conformation, with higher binding affinities to ligands compared with the homodimeric state. Interestingly, the homo-complexes of hMPC-1 and hMPC-2 showed different stabilities and it may be related to their function. In the pyruvate-transport mechanism of hMPC-1 and hMPC-2, the hetero-complex is still considered the main conformation in vivo. Inhibitors were selected according to their ability to block pyruvate uptake in vivo, in which hMPCs exist as a hetero-complex. Our study of the interaction between hMPCs and various ligands demonstrated that the heterodimeric state had a stronger binding affinity than those of the homodimeric states of hMPCs. Even if the binding affinity did not directly represent the pyruvate uptake ability, it was indicative of the transport energy barrier under the same conditions. For this reason, we hypothesized that it is significantly easier to transport pyruvate by the hetero-complex than the homo-complex, where an efficient binding pocket of inhibitors is formed by hMPC hetero-complexes. The results of our interaction studies support this hypothesis to some degree.

In summary, the changes in the homo- and hetero-conformation of hMPCs induced by differences in expression levels and stability are associated with cellular regulation, such as the compression of pyruvate uptake, the adjustment of protomer transcription, and the blocking of hMPC oligomerization, as well as disease generation and progression. Our results provide an insight into the detail properties of individual MPCs and provide a basis for further structural studies on MPCs.

## 4. Materials and Methods 

### 4.1. Construct Design

Genes encoding hMPC-2 and T-hMPC-2 were inserted into the modified pFastBac™1 (Invitrogen, Carlsbad, CA, USA) containing a hemagglutinin (HA) tag (KTIIALSYIFCLVFA), TEV cleavage enzyme recognition sequence (ENLYFQG), and 10× His tag at the N-terminus. For the T-hMPC-2, an additional sequence encoding T4 Lysozyme (T4L) was added after the 10× His tag at the N-terminus. Owing to the topological differences, the gene encoding hMPC-1 was cloned into the same vector after construct optimization containing the N-terminal HA-tag together with the PreScission enzyme recognition sequence (LEVLFQG), FLAG tag (DYKDDDDK), and 10× His tag at the C-terminus.

### 4.2. Protein Overexpression in Sf9 Cells

Plasmids generated for transposition to the Bacmid were transformed into DH10Bac™ *Escherichia coli*. Transformation procedures were performed using the Bac-to-Bac^®^ Baculovirus Expression System. Recombinant bacmid DNA of each construct (hMPC-1, hMPC-2, and T-hMPC-2) was transfection into *Spodoptera frugiperda* (*Sf9*) with Cellfectin Reagent in ESF media (Expression Systems, Davis, CA, USA) and incubated at 27 °C for 3 days. P3 virus stock (high titer) was generated from P1 and P2 virus stocks (low titers). *Sf9* cells (2 × 10^6^ cells/mL in 600 mL of biomass) were infected with the P3 virus at a multiplicity of infection (MOI: number of virus particles/number of cells) of 5 and incubated with shaking at 27 °C for 60 h before harvesting. The cultured cells were harvested, following by washing with phosphate-buffered saline (PBS), flash freezing in liquid nitrogen, and storage at −80 °C. Co-hMPC-1/-2, recombinant bacmid DNA was co-transfected into Sf9 and all other expressing process was the same as other constructs.

### 4.3. Protein Purification

Frozen cell pellets were thawed and resuspended at 4 °C until they completely melted with the addition of EDTA-free protease inhibitor cocktail (Roche, Basel, Switzerland). Then, the membrane was washed using Dounce homogenizer in hypotonic buffer containing 10 mM HEPES (pH 7.5), 10 mM MgCl_2_, 20 mM KCl and the high osmotic buffer containing 10 mM HEPES (pH 7.5), 10 mM MgCl_2_, 20 mM KCl, and 1 M NaCl, repeated three times.

The membrane fraction was then solubilized in hypotonic buffer with EDTA-free protease inhibitor cocktail (Roche) and 1% (*w*/*v*) n-dodecyl-β-D-maltopyranoside (DDM) (Anatrace, Maumee, OH, USA) at 4 °C for 3 h. The solubilized solution was isolated by ultracentrifugation at 150,000× *g* for 60 min and then incubated at 4 °C with TALON IMAC resin (Rigaku, Tokyo, Japan) overnight.

After incubation, the resin-bound hMPC-1 or hMPC-2 was loaded onto a disposable chromatography column (Bio-Rad, Hercules, CA, USA) and the resin was washed with 20 column volumes (CVs) of washing buffer containing 20 mM HEPES (pH 8), 150 mM NaCl, 30 mM imidazole, and 0.05% (*w*/*v*) DDM. Bound proteins were eluted with elution buffer containing 20 mM HEPES (pH 8), 150 mM NaCl, 500 mM imidazole, and 0.05% (*w*/*v*) DDM. The purification of untagged hMPC-2 required further reverse column application. Thus, we applied hMPC-2 to PD-10 desalting column (GE Healthcare, Chicago, IL, USA) to remove a high concentration of imidazole. Thereafter, the 10× His-tag was cleaved by TEV protease at 4 °C overnight. After the reverse affinity column, hMPC-2 was eluted in flow-through and washing step, and the target protein was collected for further analysis. In the case of hMPC-1 and T-hMPC-2, final gel filtration was performed after one-time TALON IMAC affinity chromatography. For the hMPC-1/hMPC-2 and Co-hMPC-1/-2 sample preparation, we performed additional FLAG-resin step (GenScript, NJ, USA) after TEV cleavage reaction for hMPC-2. FLAG-resin was equilibrated by buffer containing 50mM Tris-HCl (pH 7.4), 150mM NaCl, 0.05% (*w*/*v*) DDM. After the sample loading, the resin was washed with 15 column volumes (CVs) using buffer containing 50mM Tris-HCl (pH 7.4), 150mM NaCl, 0.05% (*w*/*v*) DDM. For the sample elution, we used FLAG peptide of 300ug/mL andhMPC-2 was co-eluted with FLAG-tagged hMPC-1. Final gel filtration was performed for removing FLAG peptide and change to the desired buffer. The purity of all purified proteins was checked by SDS-PAGE (10–25% gradient gel) stained by Coomassie blue. Immunoblotting was performed to confirm the hMPC-1 band on the gel using a monoclonal anti-FLAG M2 alkaline phosphatase antibody (Sigma, St. Louis, MO, USA) and WESTSAVE up (AbFRONTIER, Seoul, Korea) for visualization.

### 4.4. Nano-LC-LTQ-Orbitrap-MS

Samples were analyzed using a nano-LC system coupled with a real–hybrid equipment merged into one system (Dual cell liner trap and FT Orbitrap) (nano-LC-LTQ-Orbitrap-MS Thermo Scientific LC-LTQ Orbitrap MS installed at the National Center for Inter-University Research Facilities (NCIRF) at Seoul National University). Split-Free Nano-LC system was equipped with an EASY nLC (Thermo Scientific, US). 4 µL of the concentrated peptide extract was loaded onto a 75 µm × 15 cm column packed with 3 µm Magic C18 particles followed by a 75 min. Linear gradient from 2% to 50% ACN (*v*/*v*) in 0.1% formic acid using a 250 nL/min flow rate. The precursor ion mass tolerance was set at ± 0.8Da. Trypsin was designated as the proteolytic enzyme with up to 2 missed cleavages and treated 14–17 h. The mass spectrometer was operated in the data acquisition to automatically switch between MS and MS/MS. Survey full scan MS spectra (from m/z 15 to 4000) were acquired in the Orbitrap mass spectrometer with resolution R = 100,000 max at m/z 4000 (Mass Accuracy: <1 ppm internal calibration). Three LC-MS runs were performed for each digested sample.

### 4.5. Analytical Size-Exclusion Gel Chromatography (aSEC)

The conformational state, such as homo- and heterodimerization of hMPCs, was analyzed using a HiLoad^®^ 16/600 Superdex^®^ 200 pg column (GE Healthcare) equilibrated by aSEC buffer containing 50 mM Tris-HCl (pH 7), 300 mM NaCl, and 0.05% (*w*/*v*) DDM via ÄKTA pure (GE Healthcare). The flow rate (1 mL/min) and fraction volume (3 mL) were kept the same under all experimental conditions. The heterodimeric complex was prepared by mixing purified hMPC-1 and hMPC-2 in a mass ratio of 1:1 at room temperature for 1 h. Each fraction from aSEC was analyzed by SDS-PAGE with Coomassie blue staining and silver staining.

### 4.6. Secondary Structure and Thermal Stability Measurement

The secondary structure and thermal protein stability were monitored by circular dichroism (CD) spectroscopy. All CD experiments were performed using 0.1 mg/ mL sample in a 1-cm path length cuvette. The melting temperature (*T_m_*) of hMPCs was measured using a JASCO J-815 spectropolarimeter (Jasco, Tokyo, Japan) equipped with a Peltier system to control the temperature. Each spectrum was recorded every 5 degrees from 25 °C to 95 °C with a rate of increase of 2.5 °C/min. The CD signal around 217 nm was used to calculate the melting temperature of a protein, which attributed to the helical content.

### 4.7. Binding Affinity Analysis

A fluorescence analysis was performed using a LS55 spectrofluorophotometer at room temperature (Perkin Elmer, Waltham, MA, USA). The protein sample was prepared in buffer containing 50 mM Tris-HCl, 300 mM NaCl, and 0.05% DDM with a final concentration of 0.07 mg/ mL. The concentrations of pyruvate, UK5099, and Pioglitazone stocks were 900 mM, 100 mM, and 25 mM, respectively. Pyruvate was titrated to hMPC-1, hMPC-2, and hMPC-1/hMPC-2 up to 200mM concentrations. In the case of UK6099, it was titrated to hMPC-1, hMPC-2, and hMPC-1/hMPC-2 up to 300uM, 200uM, 200uM concentrations, respectively. Pioglitazone was titrated to hMPC-1, hMPC-2, and hMPC-1/hMPC-2 up to 400uM, 500uM, and 400uM concentrations, respectively. All data were collected in the wavelength range of 270–450 nm (shown from 300 nm to 450 nm only) with a 1-min delay for each titration point. The dissociation constants were calculated by the maximum intensities in the 338 nm wavelength of each spectrum.

### 4.8. Multiple Sequence Alignment and Homology Modeling

A sequence alignment for MPCs derived from 15 different species was generated using ClustalW and Unipro UGENE [39]. hMPC-1 or hMPC-2 was used as a reference molecule to estimate sequence similarity. Homology modeling of the monomeric state of hMPC-1 or hMPC-2 was performed using GalaxyTBM on GalaxyWEB server [40] with a bacterial SWEET transporter (PDB code: 4RNG) as a template model. The homo- and heterodimeric structures of hMPC-1 and hMPC-2 were predicted using GalaxyHomomer and GalaxyTongDock with further refinement using GalaxyRefineComplex. These programs are available on the GalaxyWEB server [40].

## Supplementary Materials

Supplementary materials can be found at https://www.mdpi.com/1422-0067/21/9/3403/s1.

## Abbreviations

hMPC	human Mitochondrial pyruvate carrier
TZD	Thiazolidinedione
PPARγ	Peroxisome proliferator-activated receptor-gamma
UK5099	α-cyano-β-(1-phenylindol-3-yl)-acrylate
GPCR	G-protein coupled receptor
TEV	Tobacco Etch Virus protease
aSEC	Analytical size-exclusion gel chromatography
SDS-PAGE	Sodium dodecyl sulfate polyacrylamide gel electrophoresis
K_d_	Dissociation constant
*T_m_*	Melting temperature
HA	Hemagglutinin
CD	Circular dichroism
DDM	n-dodecyl-β-d-maltopyranoside
*Sf*9	*Spodoptera frugiperda*
