# Supplementary material for: Characteristic Analysis of Homo- and Heterodimeric Complexes of Human Mitochondrial Pyruvate Carrier Related to Metabolic Diseases
Source: Int J Mol Sci. 2020 May 11;21(9):3403. doi: 10.3390/ijms21093403 (PMC7246999; doi:10.3390/ijms21093403)
Supplement: Supplementary file 1 [file ijms-21-03403-s001.pdf]

Table S1. Dissociation constant (K_d) of human MPCs.

	hMPC-1	hMPC-2	hMPC-1/hMPC-2
Pyruvate (mM)	11.96 ± 0.77	12.95 ± 0.90	8.914 ± 1.09
UK5099 (μM)	26.03 ± 1.47	22.46 ± 1.14	17.86 ± 0.82
Pioglitazone (μM)	84.08 ± 10.31	99.27 ± 14.52	60.26 ± 6.11

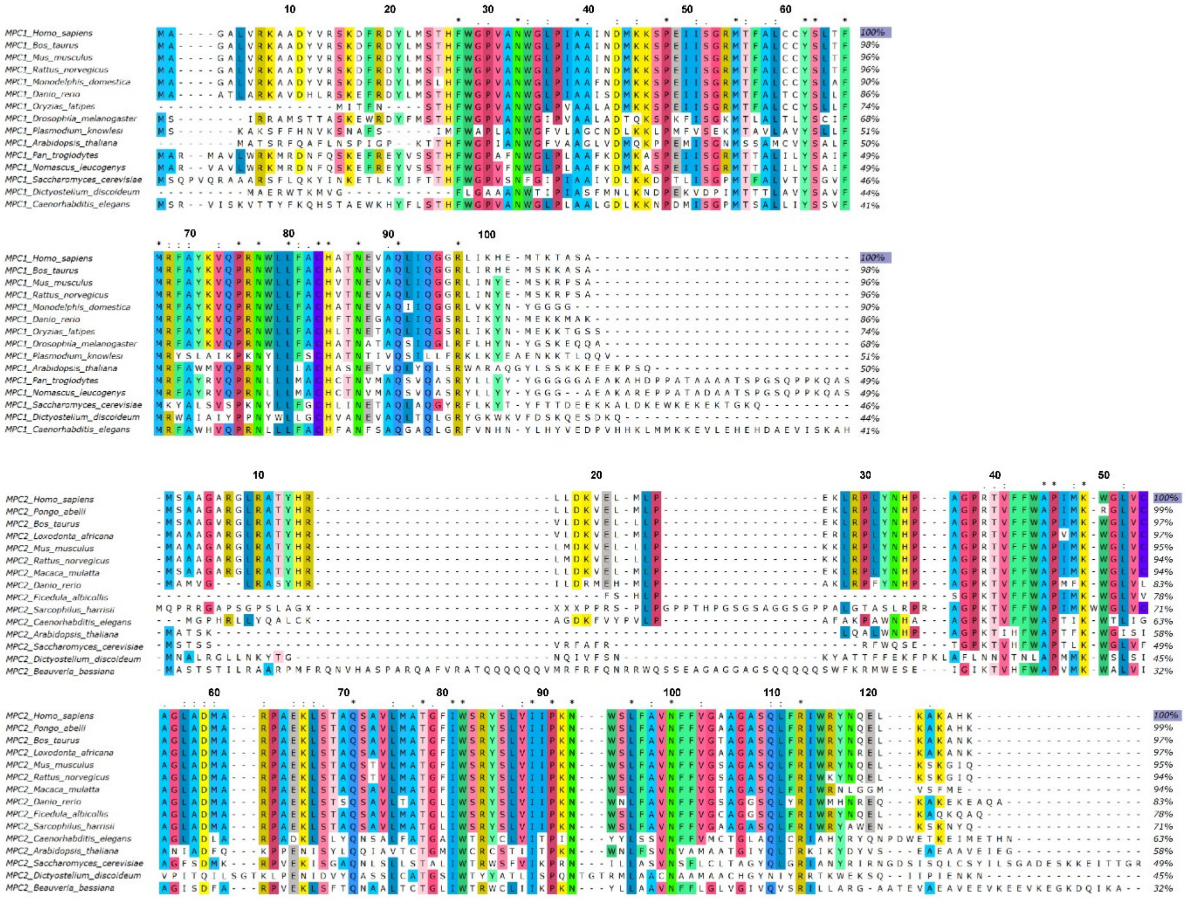

Figure S1. Sequence alignment of hMPC-1 and hMPC-2.

Protein sequence alignment of (a) hMPC-1 and (b) hMPC-2 homologs in the following species. All sequences were aligned and visualized by Clustal W and Ugene programs. Accession numbers are: Homo sapiens MPC1: NP_057182.1, *Bos taurus MPC1:* NP_001070510.1, Mus musculus MPC1: NP_061289.1, Rattus norvegicus MPC1: NP_598245.1, Monodelphis domestica MPC1: NC_008802.1, Danio rerio MPC1: NP_001002398.2, Oryzias latipes MPC1: NC_019861.2, Drosophila melanogaster MPC1: NP_001262720.1, Plasmodium knowlesi MPC1: XP_002259944.1, Arabidopsis thaliana MPC1: NP_001078606.1, Pan troglodytes MPC1: XP_001137474.1, Nomascus leucogenys MPC1: XP_003271054.1, Saccharomyces cerevisiae MPC1: NP_011435.1, Dictyostelium discoideum MPC1: XP_647090.2, Caenorhabditis elegans MPC1: NP_497894.2, Homo sapiens MPC2: NP_001137146.1, Pongo abelii MPC2: NP_001126868.1, *Bos taurus MPC2:* NP_001180050.1, Loxodonta africana MPC2: XP_003414942.1, Mus musculus MPC2: NP_081706.1, Rattus norvegicus MPC2: NP_001071111.1, Macaca mulatta MPC2: NC_041754.1, Danio rerio MPC2: NC_007120.7, Ficedula albicollis MPC2: NC_021671.1, Sarcophilus harrisii MPC2: NW_003838759.1, Caenorhabditis elegans MPC2: NP_491234.1, Arabidopsis thaliana MPC2: NP_001319940.1, Saccharomyces cerevisiae MPC2: NP_012032.1, Dictyostelium discoideum MPC2: XP_647091.1, Beauveria bassiana MPC2: XP_008602977.1.

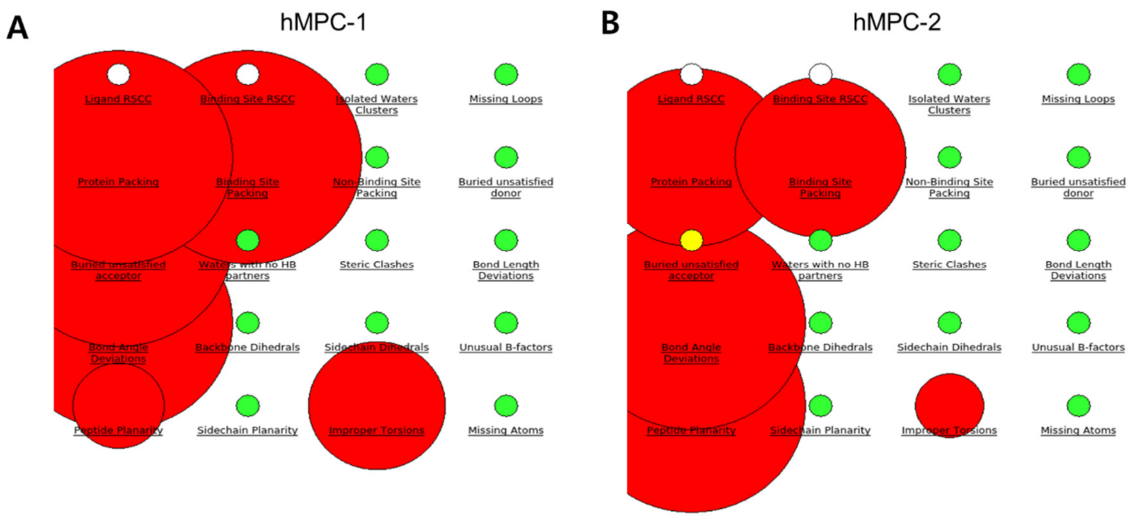

Figure S2. Structural validation of hMPC-1 and hMPC-2 model structure.

Protein packing values of (A) hMPC-1 and (B) hMPC-2 were analyzed by Maestro program.

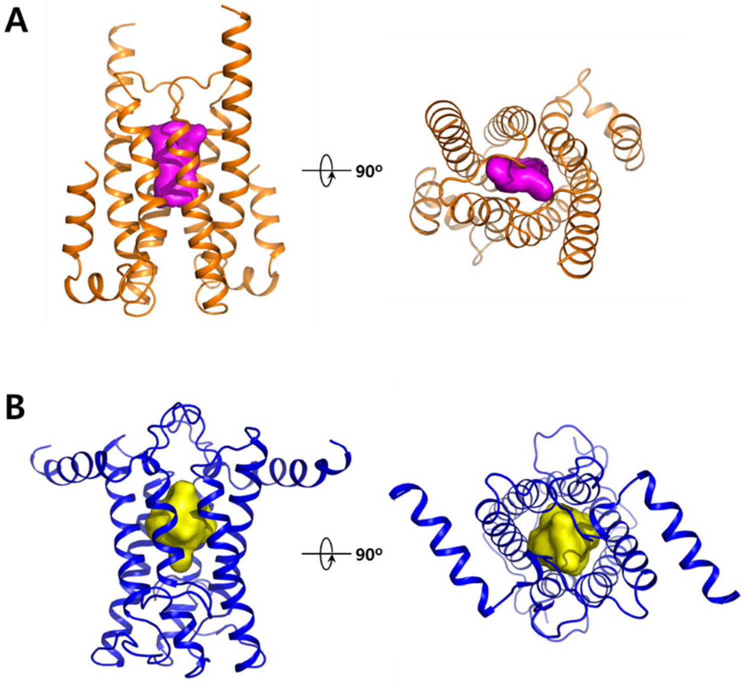

C

	Area (Å^2)	Volume (Å^3)
hMPC-1	304.188	107.106
hMPC-2	391.663	339.750

Figure S3. Analysis of the internal cavities of hMPC-1 and hMPC-2.
Analysis of the internal cavities of hMPC-1 and hMPC-2. Internal cavities of (A) hMPC-1 and (B) hMPC-2 were displayed surface model colored by magenta and yellow in the ribbon model. (C) Calculated area (Å2) and volume (Å3) of internal cavities of hMPC-1 and hMPC-2 are displayed.
